# Precision Profiling of the Cardiovascular Post-Translationally Modified Proteome

**DOI:** 10.3390/jcdd13010026

**Published:** 2026-01-01

**Authors:** Thakorn Pruktanakul, Konstantinos Theofilatos

**Affiliations:** 1 King’s British Heart Foundation Centre, King’s College London, London SE5 9NU, UK; thakorn.1.pruktanakul@kcl.ac.uk; 2 Division of Internal Medicine, Faculty of Medicine, Prince of Songkla University, Songkhla 90110, Thailand

**Keywords:** post-translational modifications, mass spectrometry, proteomics, proteoforms, bioinformatics, cardiovascular disease

## Abstract

Proteins exist as multiple chemical and sequence-specific proteoforms, each of which may serve as a critical mediator of physiological or pathological signalling. This diversity arises from processes such as alternative splicing of gene transcripts, translation into amino acid sequences, and various post-translational modifications (PTMs), leading to an exponential increase in biological complexity. This manuscript provides an overview of the mechanisms underlying proteoform generation in biological systems and highlights strategies for their analysis using mass spectrometry (MS)-based proteomics and bioinformatics. Additionally, it focuses on recent findings linking PTMs to cardiovascular disease (CVD), highlighting the MS-based methods and workflows that have been used to study uncommon PTMs and their role in CVD. This review provides a comprehensive collection of tools and knowledge to explore the breadth of proteoforms, particularly PTMs, within their specific areas of interest in cardiovascular physiology.

## 1. Introduction

Technical advancements have recently enabled the deep resolution of the human proteome in cardiovascular tissues, such as the heart [1], vascular tissues [2], and blood samples [3,4], allowing for the identification of diagnostic, prognostic, and therapeutic signatures for several conditions, such as atherosclerosis, heart failure, and coronary artery disease, clustering patients into subgroups and detecting proteins related to significant molecular mechanisms such as calcification and inflammation.

Biological diversity in systems biology is influenced by factors outlined in the Central Dogma, including DNA, RNA, and proteins [5]. While genetic variants have been extensively studied through genome-wide association studies (GWAS) and other specific studies at the tissue [3] or cell level [6], they provide only a foundational and partial explanation of biological diversity, as they do not account for the dynamic processes that regulate gene expression and protein functions.

Proteins are the primary effectors of biological processes, and understanding the variations in their forms and functions, referred to as proteoforms, provides crucial insights. This approach complements genetic studies and strengthens the systemic frameworks for investigating both physiology and pathophysiology [7]. Proteoforms arise from mechanisms, such as alternative splicing and post-translational modifications (PTMs), contributing to abnormalities across various body systems, including the cardiovascular system. For instance, metabolic remodelling is observed in the failing heart, leading to mitochondrial dysfunction and oxidative stress, which in turn induce post-translational and epigenetic modifications, ultimately activating several cellular functions [8].

Profiling and quantifying the major PTMs could serve as targets for the diagnosis and treatment of cardiovascular conditions in these participants. Previous literature has discussed computational and experimental workflows for studying clinically relevant PTMs [9,10,11,12], methods to explore the crosstalk of PTMs [13], and the related tools and resources [7]. Some reviews [14,15,16,17] have also focused on cardiovascular disease (CVD) and the PTMs, specifically significant for the function of the heart and vascular tissue, albeit with different emphases.

This review aims to explain the biological basis of proteoforms, with a focus on PTMs and their role in biological diversity; to outline mass spectrometry (MS)-based proteomics and bioinformatics strategies for PTM profiling at the proteome level; and to highlight recent findings on PTMs in the pathogenesis of CVD. Our goal is to provide cardiovascular researchers with current knowledge and state-of-the-art tools to investigate the full range of proteoforms, with particular emphasis on PTMs relevant to cardiovascular research.

## 2. Biological Basis of Proteoforms

Proteoform is an updated term for protein isoforms and is defined as the different molecular sequences and structures of protein products encoded by a single gene. They are defined by their specific amino acid sequence and any modifications, such as sequence variants, splice isoforms, and post-translational modifications [18]. Although only ~20,000 human protein-coding genes exist, the diversity of proteoforms is vastly greater, with recent studies suggesting that there are more than 1 million proteoforms in each human cell type [19].

**Figure 1:**
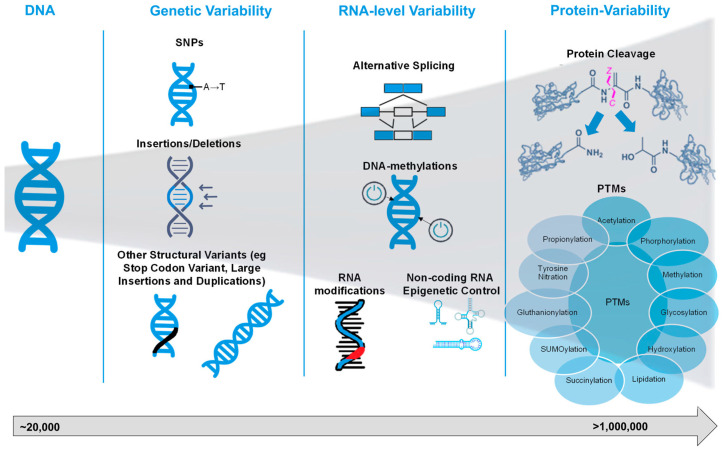
From genome to proteome and all sources of variability that end up increasing the number of proteoforms. Abbreviations: SNPs, single nucleotide polymorphisms; PTMs, post-translational modifications.

Proteoforms arise from various mechanisms at the DNA, RNA, and protein levels, which expand the functional repertoire of a single gene product. A detailed overview of the different mechanisms that might contribute to the complexity of proteoforms in humans and other organisms is summarised in Figure 1. At the DNA level, single nucleotide polymorphisms (SNPs) and other structural variants, such as duplications, insertions, deletions, frameshifts, and stop-codon variants, generate variant protein sequences that diverge from the canonical form. In dbVar [20], there are currently 8,646,824 variants reported for Homo Sapiens, but only 27,070 have been reported as pathogenic, with the remaining ones being of uncertain significance or unexplored for their clinical significance.

At the RNA level, the key mechanisms that contribute to proteoform diversity are alternative splicing and RNA modifications, which lead to an increase in the number of potential proteoforms generated from the same gene, as well as DNA methylation and non-coding RNA (ncRNAs) epigenetic mechanisms, which control which genes are transcribed to mRNA, which mRNAs are translated to proteins, and the efficiency of these processes.

Alternative splicing of pre-mRNA gives rise to multiple mRNA isoforms, which, upon translation, yield distinct proteoforms that differ in exon composition, domain structure, or protein length [21,22]. Initial studies on the effect of alternative splicing on the diversity of proteoforms, conducted using liquid chromatography tandem mass spectrometry (LC-MS/MS) analyses, revealed a surprisingly small number of alternatively spliced proteins [23]. However, recent evidence from the same group [24] has shown that when analysing tissue-specific alternative splicing at the protein level, more than a third of the studied curated alternative splicing events had significant tissue-specific differences. The importance of alternative splicing in proteoform diversity was highlighted by another study that performed ribosome profiling, which showed that approximately 75% of human mRNAs of medium to high abundance with skipped exons were detected in ribosomes [25]. This finding suggests that the importance of alternative splicing might be even more important than what was initially measured with LC-MS/MS, and this can be shown with methods that exceed the inherent limitation of low sensitivity of MS.

Epigenetic modifications and ncRNAs control the transcription of mRNA and, thus, the number of proteoforms expressed in each sample type and condition. Epigenetic modifications include secondary chemical alterations of DNA (methylation of cytosines) and post-translational modifications of histones [26], which induce changes in the state of chromatin. ncRNAs comprise diverse groups of RNAs, such as t-RNAs, small nucleolar RNAs, micro-RNAs, and long non-coding RNAs (lncRNAs) that do not encode proteins but play a fundamental role in gene expression regulatory mechanisms [27]. Recently, it has been shown that many lncRNAs might be misclassified as non-coding because they express micropeptides [28]. Moreover, a meta-analysis by Zhang et al. (2023) [29] further supported this by demonstrating that these micropeptides are functionally relevant rather than biological noise, identifying 55 with verified roles in CVD.

At the protein level, additional processes, such as proteolytic cleavage and PTMs, further diversify proteoforms. Proteolytic processing can generate truncated or activated protein forms, and PTMs (e.g., phosphorylation, acetylation, and ubiquitination) modulate the side-chain chemistry, conformation, interactions, localisation, and stability of proteoforms. PTMs are conventionally grouped into “traditional” forms, such as phosphorylation, glycosylation, methylation, acetylation, and ubiquitination, each of which is well-characterised and widely implicated in cellular regulation. For example, the modification of histone N-terminal tails by acetylation or methylation affects chromatin structure and transcriptional states. In contrast, the landscape of “emerging” PTMs has expanded rapidly in recent years to include less common—but increasingly recognised—modifications such as succinylation, crotonylation, lactylation, S-nitrosylation, and S-palmitoylation. These emerging acyl and non-canonical modifications often respond to metabolic cues, stress, or disease states and sometimes share ‘eraser’ or ‘writer’ enzymes with classical PTMs (e.g., p300/CBP acting as both an acetyltransferase and crotonyltransferase). In addition, although technically at the RNA level rather than strictly at the protein level, alternative splicing leads to isoforms that may themselves be subject to distinct PTM patterns, thus bridging the RNA-level- and protein-level layers of regulation.

## 3. MS-Based Proteomics for PTM Analysis

**Figure 2:**
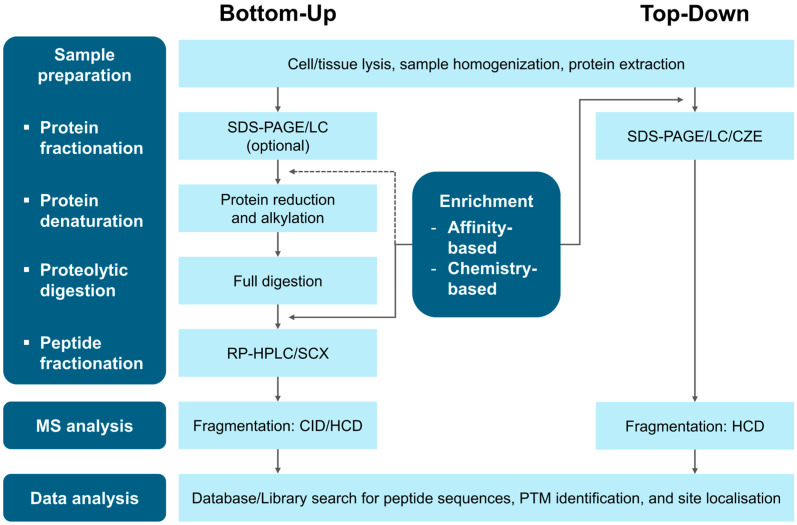
General workflows for bottom-up and top-down proteomics with commonly used methods. Abbreviations: CID, collision-induced dissociation; CZE, capillary zone electrophoresis; HCD, higher-energy collisional dissociation; LC, liquid chromatography; MS, mass spectrometry; RP-HPLC, reverse-phase high-performance liquid chromatography; SDS-PAGE, sodium dodecyl sulphate polyacrylamide gel electrophoresis.

MS-based proteomics is a highly sensitive and specific analytical technique for investigating proteins and their PTMs in biological samples. It is recognised as the gold standard for protein and PTM analysis and enables the identification, localisation, and quantification of a wide range of PTMs [30]. Bottom-up, middle-down, and top-down proteomics approaches are used for PTM analysis. However, middle-down proteomics is primarily employed to study specific PTMs or PTM crosstalk in purified proteins [13], limiting its applicability. Therefore, this review focuses on bottom-up and top-down approaches (Figure 2), along with the essential steps in PTM analysis, including enrichment methods and fragmentation modes.

### 3.1. Bottom-Up Proteomics Workflow

Bottom-up proteomics identifies and quantifies proteins and their PTMs by analysing proteolytically derived peptides [31]. The workflow begins with protein extraction from biological samples, followed by proteolytic digestion to generate the peptides. To enhance the detection of low-abundance PTMs, an enrichment step is often incorporated before or after digestion [30]. The resulting peptides are then separated by LC, ionised, and introduced into the MS, where their mass-to-charge ratios (*m*/*z*) are measured to generate MS spectra. Precursor ions are fragmented to generate product ions, yielding MS/MS spectra for peptide identification and protein inference. Protein quantification is typically performed by spectral counting or by calculating the area under the curve of MS chromatograms. The impact of enrichment and fragmentation methods on PTM analysis is discussed later.

Incorporating PTM-specific enrichment increases the risk of technical variability, potentially confounding the biological interpretations [17]. Label-based strategies mitigate this issue by enabling sample multiplexing and reducing variance during preparation [32]. Label-based methods generally lack specificity for modified proteins; thus, certain approaches, such as iodoTMT labelling for S-nitrosylation and sulfenylation or thiol-based labelling [33], have been developed to target specific modifications.

### 3.2. Top-Down Proteomics Workflow

Top-down proteomics characterises intact proteins by bypassing the digestion step of bottom-up workflows. Instead, proteins are fractionated at the proteoform level to facilitate their global characterisation [34]. MS spectra provide *m*/*z* ratios of intact proteins, while MS/MS spectra reveal large fragment ions, enabling the precise identification, localisation, and analysis of combinatorial PTM patterns on individual proteoforms [35].

Despite its advantages, top-down proteomics faces challenges, such as limited sensitivity and throughput, potentially leading to undetected low-abundance proteoforms and ambiguous PTM site localisation. Furthermore, overlapping isotopomer envelopes in large-fragment ions create complex spectra, necessitating deconvolution algorithms [17]. These algorithms resolve the complexity of producing monoisotopic masses, charge states, and intensities, which are then matched against databases for protein identification, akin to peptide analysis in bottom-up workflows.

### 3.3. PTM Enrichment Methods

PTM enrichment methods are integral to proteomics workflows, enabling the selective isolation of modified proteins or peptides from unmodified ones. These methods reduce sample complexity and enhance the sensitivity and specificity of downstream MS analysis for specific PTMs’ identification. Enrichment strategies are broadly categorised into affinity-based and chemistry-based approaches [30].

Within affinity-based approaches, chromatographic enrichment methods exploit the physicochemical properties of PTMs, such as affinity to metal species, charge, and hydrophilicity, to enable selective isolation prior to MS analysis [30]. Examples include immobilised metal ion affinity chromatography (IMAC), which leverages the affinity of negatively charged phosphate groups for positively charged metal ions such as Fe^3+^ and Ga^3+^ and metal oxide affinity chromatography (MOAC), which uses metal oxides such as TiO_2_ to bind phosphate groups. Strong cation exchange (SCX) chromatography captures positively charged peptides under acidic conditions, enabling the early elution of highly negatively charged PTMs. Strong anion exchange (SAX) chromatography employs positively charged resins to enrich negatively charged PTMs. Hydrophilic interaction liquid chromatography (HILIC) separates PTMs based on hydrophilicity using a hydrophilic stationary phase and a hydrophobic organic mobile phase. These techniques can be used individually or in combination for more efficient enrichment; however, they are generally less effective at isolating low-abundance PTM subclasses, such as phosphotyrosine, compared with more abundant modifications such as phosphoserine or phosphothreonine.

In addition to chromatographic methods, affinity-based enrichment also encompasses biological affinity reagents, including antibodies and PTM-binding domains, which rely on specific molecular recognition. Antibody-based immunoprecipitation enables highly PTM-specific enrichment, exemplified by anti-phosphotyrosine antibodies [36]. Alternatively, protein-binding domain pull-down methods offer higher affinity and broader coverage for specific PTMs [37]. Despite their utility, antibody-based methods face challenges such as limited antibody availability, non-specific binding, and motif bias, often requiring antibody mixtures for comprehensive enrichment [30].

Chemistry-based methods overcome some of the limitations of antibody-based methods. They involve tagging modified peptides or proteins with biotin using chemoselective probes, metabolic labelling with unnatural precursors, or chemoenzymatic approaches, followed by streptavidin-based enrichment [38]. The strong biotin-streptavidin interaction ensures the retention of target PTMs during washing.

### 3.4. Fragmentation Methods

The choice of fragmentation method is critical in proteomics, as it influences the analysis of labile PTMs and the accuracy of peptide sequencing. Collision-induced dissociation (CID) is commonly used in bottom-up proteomics and generates b- and y-ion fragment series [39] for peptide sequencing and PTM localisation. However, labile PTMs such as phosphorylation and glycosylation are prone to neutral loss during fragmentation, potentially obscuring PTM site identification [40]. In contrast, stable PTMs remain attached, resulting in predictable mass shifts.

Higher-energy collisional dissociation (HCD) operates at higher collision energies than CID, often producing more comprehensive backbone fragmentation than CID. Although it can also cause neutral losses of labile PTMs, it generally provides better sequence coverage [41], aiding PTM site identification.

Electron transfer dissociation (ETD) is well suited for preserving labile PTMs during fragmentation. By generating c- and z-ions without imparting excessive vibrational energy, ETD maintains the integrity of labile modifications [42]. It provides both peptide sequence information and diagnostic ions that aid in PTM localisation [43]. Consequently, ETD is widely used in top-down proteomics and is increasingly applied in bottom-up workflows focused on PTM analysis.

By employing appropriate strategies according to the research aims, proteomics workflows can achieve higher sensitivity, specificity, and reproducibility in PTM analysis, facilitating their application in cardiovascular proteomics, as summarised in Table 1.

## 4. Bioinformatics in PTM Analysis

The computational analysis of protein PTMs involves a range of tasks, including identifying modifications, localising modification sites, quantifying PTMs, assessing stoichiometry, visualising molecular networks, and validating PTM assignments and functions. A wide array of bioinformatics tools has been developed to address these tasks, with some designed for specific purposes and others integrating multiple functions. In this section, we focus on key PTM tools, including databases and tools for PTM identification and localisation.

### 4.1. Databases for PTM

Many tools offer PTM databases, which we have categorised into three groups based on their distinct purposes and features. The first group comprises PTM-specific databases such as PhosphoSitePlus [51], which covers multiple PTM types across a wide range of species. Many of these databases focus on specific PTM types, including resources such as O-GlycBase [52] for O- and C-linked glycosylation, UniCarbKB [53] for carbohydrate modifications, UbiProt [54] for ubiquitination, and phosphorylation-focused databases, such as PHOSIDA [55], Phospho.ELM [56], PhosphoPep [57], and PhosPhAt [58]. The second group includes curated comprehensive databases, which are general protein databases containing curated PTM information within broader entries. Examples include UniProtKB [59] and neXtProt [60]. The third group consists of integrative databases, such as PTMcode2 [61], dbPTM [62], and iPTMnet [63]. These databases combine data from various sources and utilise predictive algorithms to provide insights into PTM interactions, structural contexts, and functional crosstalk.

In addition to these categories, the ProteomeTools [64] project has leveraged a large collection of synthetic peptides to analyse the multimodal LC-MS/MS characteristic of essential human peptides, including those with important PTMs. Although not a traditional PTM database, ProteomeTools offers valuable experimental data related to PTMs. Table 2 presents the major PTM-related databases and their key features, with further details on iPTMnet and ProteomeTools provided in the subsequent discussion.

iPTMnet is an integrative and predictive database. It serves as an online knowledge discovery platform, offering comprehensive PTM functionality, including searching, browsing, visualisation, and network exploration [63]. The platform supports eight major PTM types: phosphorylation, ubiquitination, acetylation, methylation, glycosylation, S-nitrosylation, sumoylation, and myristoylation. The PTM data in iPTMnet are sourced from text mining PubMed using RLIMS-P and eFIP tools, curated experimental databases, and the Protein Ontology, which provides an ontological representation and comparison of PTMs across species. Updated monthly, the knowledgebase enables interactive queries and PTM network visualisation. However, the current version is limited in its text mining capabilities, as only protein names confidently linked with database identifiers are displayed, restricting the full utilisation of available data.

ProteomeTools offers insights into the multimodal LC-MS/MS characteristics of 21 essential modifications for bottom-up proteomics [64]. The project utilises approximately 5000 synthetic peptides to investigate properties such as retention time shifts, precursor charge state variations, and changes in search engine scores associated with specific modifications. In addition to confirming 10 known diagnostic ions for lysine modifications, this study identified five novel ions through systematic investigation. The data generated in this project are available on the ProteomeTools website.

### 4.2. Tools for PTM Identification and Localisation

#### 4.2.1. Bottom-Up Proteomics

In bottom-up proteomics, general search engines such as Mascot [65], SEQUEST [66], Andromeda [67], and MSFragger [68] identify peptide sequences and potential PTMs from MS/MS spectra using approaches such as sequence search, spectral library search, or hybrid methods [69]. However, while these search engines typically provide confidence scores for peptide ions, they often lack robust scoring methods for PTM site localisation [70], making precise modification site assignment challenging.

To address this limitation, specialised PTM localisation tools are frequently used alongside search engines. These tools reanalyse MS/MS data to refine PTM localisation and calculate confidence scores or probabilities for site assignments. Many such tools exist, most of which are tailored to specific search engines or PTM types. For instance, ASCORE [71] estimates localisation probabilities for phosphopeptides identified by SEQUEST or Mascot, whereas the PTM Score algorithm reprocesses Andromeda search results within the MaxQuant software.

Newer search engines with built-in PTM analysis, such as Byonic [72], and newer post-search refinement tools compatible with widely used search engines, such as PTMProphet [70] and MetaMorpheus [73], enhance localisation accuracy and analysis efficiency while supporting a broader range of PTMs.

In Table 3, we provide a list of the major computational tools for the identification, quantification, and analysis of post-translational modified proteins, highlighting their features and PTM-specific applications. Further details of Byonic, PTMProphet, and MetaMorpheus are provided in this section.

Byonic is a commercial search engine for comprehensive peptide and protein identification, integrated within the Proteome Discoverer environment [72]. It offers three key features: modification fine control, wildcard search, and glycopeptide searches. Modification fine control enables users to adjust search parameters, reducing the risk of omitting relevant peptides and minimising false positives caused by overly broad searches. Wildcard search facilitates a single search for both unanticipated and novel modifications within each peptide fragment. Glycopeptide searches identify glycosylated peptides without the need for predefined glycosylation sites or glycan masses. These capabilities enable the simultaneous identification of up to hundreds of modification types while minimizing the combinatorial complexity.

PTMProphet, a free, open-source tool within the Trans-Proteomic Pipeline [86], helps overcome these limitations by supporting various commonly used search engines. It also reanalyses search results to assess modification localisation confidence for any PTM type. PTMProphet calculates robust metrics, such as the local false localisation rate (FLR), which represents the probability of accurate assignment for individual potential modification sites, and the global FLR, which evaluates the overall confidence across all identified sites at a given threshold [70]. These probabilities are provided in a standardised pepXML output format, enabling comprehensive PTM analyses.

MetaMorpheus improves global PTM discovery by enhancing search efficiency and specificity [87]. This improvement was achieved by replacing the initial open search with a multi-notch search, which provides greater specificity while reducing computational demands. Additionally, the modification list has been significantly expanded to include a broader range of biologically important PTMs during database augmentation. In the final search step, the traditional narrow-window search was replaced with a limited multi-notch search to address precursor mass deisotoping errors and facilitate the identification of co-isolated peptides. These advancements have increased the number of confidently identified peptides and PTMs.

While current tools perform well for PTM identification and site localisation, challenges remain in quantitative interpretation, particularly regarding the normalisation of PTM signals against total protein abundance. This remains a prerequisite for stoichiometry-aware comparisons across samples [88]. Most approaches treat PTMs as independent events, limiting insight into combinatorial crosstalk and hierarchical regulation [33]. Additionally, current bioinformatics pipelines are primarily optimised for bulk proteomics [70,71,72,73], with limited adaptation for single-cell, spatial, structural, or clinical contexts.

#### 4.2.2. Top-Down Proteomics

Unlike bottom-up proteomics, top-down proteomics overcomes the challenge of detecting modifications at distant sites on the same protein by analysing intact proteins, preserving the full PTM context. This approach generates complex *m*/*z* spectra, which necessitate spectral deconvolution and data processing prior to database searching. Search engines designed for top-down proteomics, such as ProSight PD, TopPIC [83], pTop [84], and MSPathFinder [85], are optimised to handle these challenges and facilitate the identification of PTMs in intact proteins.

ProSightPD is an adapted version of the ProSight PTM algorithm [89], optimised for integration into the Proteome Discoverer software. It employs a Poisson match-scoring model that leverages protein annotations to reduce the extensive search space typically encountered in top-down proteomics identification. The MS/MS spectra were deconvolved using the Xtract algorithm, and cRAWler processed the raw data to generate input for ProSightPD. The analysis utilised the UniProtKB database in XML format to provide comprehensive protein sequence information and annotations.

Another three tools—TopPIC, pTop, and MSPathFinder—utilise a spectral alignment algorithm that aligns deconvolved MS/MS spectra with sequence-derived mass ladders by interpreting PTM-containing regions as unexplained mass differences. In TopPIC, proteoform identification is the primary function, encompassing protein filtering, spectral alignment, and computation of E-values for proteoform-spectrum matches (PrSMs) [83]. The software also provides an optional module for characterising unknown mass shifts in PrSMs using the MIScore method, which applies Bayesian modelling. Because accurate spectral alignment depends on high-quality deconvolution, TopPIC relies on the companion TopFD tool to generate deconvolved MS/MS spectra.

pTop improves search speed, particularly for proteins with multiple PTMs, by utilising sequence tags derived from MS/MS spectra along with a dynamic programming algorithm [84]. To further enhance precursor deconvolution, a machine-learning method, specifically a support vector machine, was integrated into the pParseTD module. In the Informed Proteomics Suite, MSPathFinder performs database searches using deconvolved LC-MS spectra processed by ProMex, which can detect LC-MS features with high accuracy [85].

Despite the availability of advanced PTM identification and localisation tools for both bottom-up and top-down workflows, community-wide benchmarking standards for localisation accuracy across different tools, PTM types, and experimental platforms remain limited [90], highlighting the need for standardised datasets and validation strategies.

## 5. PTMs in Cardiovascular Diseases

PTMs occur at the C- or N-termini of proteins, as well as on specific amino acid side chains. These modifications encompass a wide spectrum of molecular alterations, ranging from small covalent chemical modifications to the conjugation of proteins or macromolecules. PTMs can be broadly classified as reversible, involving covalent modifications such as phosphorylation or acetylation, or irreversible, involving proteolytic processing events. Moreover, PTMs may arise through enzymatic, chemical, or physical mechanisms [91].

Commonly studied PTMs include phosphorylation, acetylation, ubiquitylation, methylation, glycosylation, sumoylation, palmitoylation, myristoylation, prenylation, and sulfation, and their role in CVDs, such as heart failure, coronary artery disease, myocardial infarction, vascular calcification, and atherosclerosis, have been widely studied in previous review manuscripts [14,16,92,93]. In summary, some of the key discoveries related to common PTMs, the role of protein arginine methyltransferases (PRMTs) in the methylation of arginine residues and other factors regulating cholesterol synthesis and efflux and being involved in atherosclerosis and heart failure [94], acetylation, and deacetylation of functional proteins have been shown to be essential for the homeostatic regulation of embryonic development, postnatal maturation, cardiomyocyte differentiation, cardiac remodelling, and onset of various CVDs [95], while protein glycosylation regulates diverse homeostatic functions, such as contractility, metabolism, transcription, and signalling pathways, contributing to the pathophysiology of heart failure and the cardiovascular system [96].

Despite the significant findings for commonly studied PTMs, recent evidence has shown that the diagnosis, prognosis, and treatment of many cardiovascular disorders can be facilitated with non-commonly studied PTMs such as carboxylation, succinylation, S-nitrosylation, malonylation, and different types of oxidation [97]. In this context, Bagwan et al. (2021) [98] were the first to perform a proteome-wide profiling and mapping of post translational modifications in human hearts, quantifying 150 PTMs in human hearts using a combination of COMET-PTM, SHIFTS, PTM-sticker, and PTM-Shepherd tools. In this section, we focus on the association of non-common PTMs with CVDs and their study using MS-based proteomics workflows. Table 4 presents exemplary studies in the last five years exploring the role of uncommonly studied PTMs in CVDs using MS-based proteomics in human and animal models with more than 10 samples.

Gamma carboxylation describes the post-translational modification in which glutamate residues are replaced with -carboxyglutamate, forming Gla residues. This modification is facilitated by the enzyme -glutamyl carboxylase and the coenzyme vitamin K. Proteins can have domains containing large numbers of Gla residues, which are thus known as Gla proteins. Some of these proteins are related to atherosclerosis and CVDs through their ability to control vascular calcification. Some major γ-carboxylated proteins are matrix Gla protein (MGP) and Gla-rich protein (GRP). MGP is the γ-carboxylated and thus activated form of matrix glutamate protein, which is synthesised by vascular SMCs. The method by which MGP regulates calcification lies in its high affinity for calcium ions relative to its inactive form, allowing it to bind free calcium and thus prevent the aggregation and subsequent mineralisation of calcium ions. MGP also regulates matrix mineralisation by regulating processes related to SMCs which contribute to calcified phenotypes [106]. MGP exists in various forms, and the active form must be both carboxylated and phosphorylated. Therefore, the phosphorylation of MGPs serine residues, a step dependent on vitamin K, is critical for MGPs’ efficacy in inhibiting calcification [107]. GRP, similarly to MGP, is activated through -carboxylation and prevents vascular calcification by modulating crystal growth in the extracellular matrix [108]. The Proteomic Atlas of Atherosclerosis has recently identified a signature of gamma carboxylated proteins, including MGP, Thrombin (F2), growth arrest-specific protein (GAS6), Vitamin K-Dependent Protein C (PROC), and Vitamin K-Dependent Protein Z (PROZ), which were upregulated in calcified and asymptomatic plaques from carotid endarterectomies [2].

Lysine succinylation is an MS-identified high-abundance PTM [109] that induces significant structural changes in the proteoforms of significant proteins, such as SERCA2, which are associated with heart failure. Yang et al. (2025) [99] showed that Succinylation of SERCA2a at K352 promotes its ubiquitinoylation and degradation by proteasomes in sepsis-induced heart dysfunction. To further explore the functional role of this PTM, they combined co-immunoprecipitation with LC-MS/MS and identified that SIRT2, a deacylase, interacted with SERCA2a and that SIRT2 decreased K352 succinylation of SERCA2a, suggesting that SIRT2 may function as a desuccinylase and controller of this PTM for SERCA2a. Furthermore, Zhang et al. (2023) [110] studied global lysine succinylation using western blots and found that it was significantly increased in patients with thoracic aortic aneurysm and thoracic aortic dissections with pre-existing aortic aneurysms compared with healthy subjects. Among other succinylation-affected proteins, using tandem mass tag (TMT)-labelled LC-MS/MS, OXCT1 was found to be significantly upregulated and thus suggested as a novel marker of thoracic aortic aneurysm.

S-nitrosylation is a PTM characterised by the reversible, covalent addition of a Nitric Oxide moiety to the thiol group of the cysteine residues of proteins [111]. Li et al. (2025) [100] applied an S-nitrosylation-specific workflow to study the S-nitrosylated proteins associated with heart failure with a preserved ejection fraction. In particular, S-nitrosylated proteins were labelled and isolated using a Pierce S-nitrosylation Thermo Scientific labelling kit and then analysed using an established TMT-based quantitative proteomic workflow [112]. The main proteomic findings of this study included a clear segregation between the S-nitrosylated proteomes of ZSF1 obese rats and Wistar Kyoto (WKY) control hearts, indicating a marked global shift in nitrosylation patterns in the HFpEF context. ZSF1-specific and additional dysregulated S-nitrosylated proteins were identified, and the main findings were validated using western blots and cross-analysed in combination with single-nuclei RNA-sequencing data. In smaller scale studies, S-nitrosylation has also been associated with atherosclerosis [113], regulation of oxidative stress [114], and other metabolic pathways that are significant for heart function [115].

Lysine malonylation (Kmal) requires malonyl-coenzyme A(CoA) to modify lysine, switching its charge from positive to negative and altering the protein structure [116]. Wu et al. (2022) [101] applied label-free LC-MS/MS profiling of KMal in mice hearts after sham and TAC operations, using a MaxQuant-based workflow, and identified malonylated proteins enriched in cardiac structure and contraction, the cGMP-PKG pathway, and metabolism. Moreover, among the 172 malonylated sites and 150 consistently quantified malonyl peptides in 87 proteins, 5 sites in 2 proteins were dysregulated in cardiac hypertrophy, and 17 sites in 13 proteins were only expressed in the control samples. The key finding validated with low-throughput experiments was the downregulation of IDH2 malonylation and its association with cardiac hypertrophy. In the context of myocardial infarction, malonylation was studied in human serum samples together with succinylation and glutarylation using LC-MS/MS identifying albumin as the most modified proteins in ST-segment elevation myocardial infarction human and rat serum samples [117].

Yang et al. (2023) [102] further studied malonylation, together with other non-commonly studied modifications (e.g., lysine β-hydroxybutyrylation) in heart samples of naturally senescent mice, using a MaxQuant-based label-free LC-MS/MS proteomics workflow. The key findings of the study were related to lysine β-hydroxybutyrylation (Kbhb) a novel PTM in which β-hydroxybutyrate (βHB) is covalently attached to lysine ε-amino groups [118]. In the cardiac tissue of young and aged mice, 1710 β-hydroxybutyrylated lysine sites in 641 proteins were identified in the cardiac tissue of young and aged mice with 183 Kbhb sites identified in 134 proteins exhibiting significant differential modification in aged hearts. The Kbhb-modified proteins upregulated in the hearts of aged mice were primarily detected in energy metabolism pathways and localised in the mitochondria. Another study from the same group [103] examined the Kbhb profile of vascular tissues from healthy individuals and patients with metabolic syndrome-induced restenosis using the same workflow, with one of the main findings being the association of Kbhb modification of COL1A1 with disease pathogenesis.

Lactylation is a PTM that can occur on lysine residues of both histones and non-histone proteins, directly regulates gene expression and protein function, and plays a significant role in ischaemia-reperfusion injury [119]. Using a label-free LC-MS/MS MaxQuant-based workflow, Wang et al. (2025) [104] analysed the lactylome of mouse myocardium during ischaemia-reperfusion. The major finding was that Serpina3k and its lactylation at lysine 351 increased upon reperfusion. This finding was further explored in a functional study overexpressing the lactylation-deficient mutant in Serpina3k knockout mice, demonstrating the protective role of Serpina3k and Serpina3 lactylation through their secretion from ischaemia-reperfusion-stimulated fibroblasts to protect cardiomyocytes from reperfusion-induced apoptosis. Further bioinformatics meta-analysis has recently associated lactylation of histones and other proteins with atherosclerosis and other CVDs [120].

Oxidation plays a key role in the initial formation of fibrotic lesions in atherosclerosis. This is observed through LDL oxidation and free radical formation, eliciting a pro-inflammatory response [121]. Specifically, ox-LDL not only causes the recruitment of macrophages to the tunica intima but also acts as a ligand for macrophage scavenger receptors, allowing the molecule to easily become internalised into the macrophages. The accumulation of ox-LDL in macrophages forms the so-called ‘foam cells’. When foam cells die, they release their stored ox-LDL into the surroundings, which nearby macrophages then internalise in a cycle. This process eventually forms an initial lesion. This lesion forms around its oxidised centre and then progressively forms a plaque through the addition of calcification and the accumulation of collagen and foam cells [122]. Overall, similar to gamma-carboxylation, oxidation is a key factor in initiating atherosclerosis, and oxidised elements such as foam cells are localised in the structure of the plaque. Therefore, an analysis of oxidation is relevant to the investigation of the disease. Oxidation of proline, lysine, and methionine is usually used as a dynamic modification in most proteomic studies on cardiovascular disorders. However, the oxidised peptides identified from such studies are not studied separately to identify the molecular mechanisms involved. Hasman et al. (2023) [105] analysed oxidised peptides separately from unmodified peptides and co-expression networks were reconstructed and analysed using label-free proteomics of carotid endarterectomy samples. They revealed that the unique interactors of oxidised FLNA were enriched in cellular responses related to cell–cell communication, neutrophil degranulation, and smooth muscle cell contraction.

## 6. Discussion and Conclusions

Proteins exist as multiple chemical and sequence-specific proteoforms in the human body. Their diversity is affected by many factors at the DNA, RNA, and protein levels; however, PTMs are the most important factor at the protein level. This manuscript reviews the biological basis of PTM-produced proteoforms, outlines MS-based proteomics and bioinformatics strategies for PTM profiling in the proteome, and highlights recent findings on PTMs in CVD pathogenesis. Emphasis was given to uncommonly studied PTMs that have recently been shown to be important for various cardiovascular disorders and the aging heart and vasculature.

Databases and tools for the analysis and study of PTMs are presented in detail, presenting their features and discussing their availability. However, to date, there has been no comprehensive benchmarking study assessing the performance of computational workflows for performing PTM-specific searches on MS data. The generation of well-studied benchmarking data from recently published studies could be the first step in this direction.

Most applications for the study of PTMs in cardiovascular-related tissues and biofluids have been performed using standard LC-MS/MS labelled and label-free workflows, but they have been applied to PTM-enriched samples using immunoprecipitation or other PTM enrichment methods. Despite the development of many computational workflows [67,70,72,73] for an expanded search of PTMs, this has not been feasible, at least for low-abundance ones. Including additional variable modifications substantially increases the search space, reducing the sensitivity of the overall approach and making a wide search for PTMs impractical [123,124]. Thus, PTM searches should be driven by hypotheses and involve targeted searches for specific modifications of interest. With the improved sensitivity of mass spectrometers, wider searches will be enabled, and the bioinformatics tools that support them will become increasingly relevant.

Recent advances in the development of large language models and artificial intelligence have begun to enable new research avenues for the study of PTMs [125]. In particular, two prominent lines of research focus on the identification of potential PTM sites in proteins [126,127,128] and on methods for characterising the downstream functional sequences of PTMs, including their implications for drug design [126,129]. Most existing approaches harness the power of AI to mine and integrate knowledge from published literature; however, this information is, in most cases, limited to PubMed abstracts. The incorporation of data from proteomics repositories, such as PRIDE, alongside the development of customised AI models capable of utilising these resources, would enable exploration of human protein isoforms with substantially greater coverage.

Additional advances in proteomics, including single-cell and spatial proteomics workflows, offer further opportunities for studying PTMs at single-cell or spatial resolution. Although single-cell and spatial proteomics approaches have already been applied to investigate protein-level changes in cardiovascular diseases [130,131,132,133], their current sensitivity remains limited, restricting the detection of low-abundance PTMs. Nevertheless, as demonstrated by Mun et al. [134] and Orsburn et al. [135], single-cell proteomics can be used to investigate PTM heterogeneity across cell states and signalling dependencies, as well as to reveal epigenetic drug-induced changes at the level of individual nuclei. Furthermore, Bagwan et al. [98] showed that PTMs exhibit substantial heterogeneity across different chambers of the human heart. Thus, the development of more sensitive single-cell and spatial proteomics methodologies could enable the identification of cell- and tissue-specific PTMs that are highly associated with pathophysiological mechanisms in cardiovascular disease.

One important limitation of existing bioinformatics workflows is the lack of available workflows for reconstructing PTM-specific networks. As illustrated by Hasman et al. (2023) [105], modified proteins have different interaction partners, which is expected because PTMs affect protein structure. Sarohi and Basak (2023) [136] also employed this approach to develop a comprehensive site-specific collagen PTM map of COL1A1 for stent-induced neointima formation. Similarly, Ma et al. (2025) [137] deployed Weighted Gene Co-expression Network Analysis (WGCNA) to identify lactylation-specific co-expression networks and study the mechanisms of atrial fibrillation. However, these are only exceptions, and most studies that reconstruct molecular networks from cardiovascular and other tissues treat modified and unmodified proteins as the same entity, aggregating their abundances. Thus, the development of novel network reconstruction techniques is required to enable the analysis of modified and unmodified proteins in the same workflow.

One of the major strengths of network-based approaches is their ability to efficiently and meaningfully integrate multi-omics data. This principle is equally relevant to the study of PTMs, where network-based approaches can combine quantitative omics modalities, such as RNA-sequencing, LC-MS proteomics, and metabolomics [138]. For the investigation and characterisation of PTMs in particular, the integration of transcriptomics with LC-MS proteomics and metabolomics is essential, as it enables a deeper understanding of PTM functionality by capturing the complementary and combinatorial effects of gene expression, protein abundance, and metabolite levels. Using sequential, cell-level integrated proteomics and metabolomics data from cultured human pluripotent stem cell–derived cardiomyocytes, Bayne et al. [139] identified overrepresented pathway networks involved in protein synthesis, oxidative phosphorylation, and cardiac muscle contraction. Similarly, Contessoto et al. [140] combined transcriptomics, proteomics, and glycoproteomics data to identify and characterise distinct molecular profiles in non-ST-segment elevation myocardial infarction. More advanced multi-omics workflows have recently been developed for cancer research [141,142], with a particular focus on proteoform characterisation, and the adaptation of similar technologies and analytical strategies to cardiovascular diseases represents a promising future research direction. Ultimately, the goal should be the comprehensive characterisation of multi-omic networks at both tissue and cell levels, analogous to achievements reported in certain plant types [143].

From a future-oriented perspective, important fields of research include the “PTM-crosstalk” concept, where multiple PTMs on a single protein or network act in concert or in competition to regulate function. Moreover, the expansion of PTM types into previously under-explored PTMs, such as γ-carboxylation and lactylation, can provide insight into the mechanisms of heart failure, atherosclerosis, and other CVDs. Finally, the translation of some of the identified PTM-specific biomarkers into diagnostic and prognostic biomarkers or novel therapeutic targets is promising. The more sensitive and accurate quantitative detection of low-abundance PTMs in patient tissues or plasma and network-based modelling of cardiovascular pathophysiology remain key challenges. As technology continues to evolve, the systematic characterisation and functional interrogation of PTMs in cardiovascular cells and tissues will likely yield novel diagnostic markers and therapeutic interventions across the spectrum of CVD.

## Figures and Tables

**Table 1:** Comparative overview of proteomics strategies for PTM analysis in cardiovascular research.

Strategies	Workflow Applicability	Strengths	Limitations	Suitability for CV Tissues and Plasma	Primary Applications
Proteomics workflows					
Bottom-up proteomics	CID/HCD (standard); ETD (for labile PTMs)	Scalability; depth; compatibility with di-verse PTMs	Loss of proteoform context; PTM crosstalk inference	High: Standard for deep tissue and plasma mapping	Global PTM mapping and comparative profiling [44,45]
Top-down proteomics	ETD (preserves PTMs on large proteins)	Preserves combinatorial PTMs and proteoform integrity	Limited coverage; complex data analysis	Moderate: Suitable for high-abundance proteins	Proteoform-level characterisation and PTM co-occurrence analysis [46,47]
PTM enrichment methods					
Affinity-based chromatographic enrichment	Bottom-up (predominant); top-down (feasible)	Exploit PTM physicochemical properties; scalable; combinable	Less effective for low-abundance PTM subclasses	High: Well-suited for major PTM classes	Global enrichment of major PTM classes [48]
Affinity-based biological enrichment	Bottom-up (predominant); top-down (feasible)	High specificity; effective for low-abundance PTMs	Motif bias; limited availability; non-specific binding	High: Well-suited for low-abundance PTMs	Targeted enrichment of specific PTM classes [49]
Chemistry-based enrichment	Bottom-up (predominant); top-down (feasible)	Chemoselective, covalent capture; strong enrichment efficiency	Requires PTM-specific chemistry; increased experimental complexity	Moderate: Well-suited for labile or rare PTMs	Detection of chemically tractable or labile PTMs [50]

Abbreviations: CID, collision-induced dissociation; CV, cardiovascular; ETD, electron transfer dissociation; HCD, higher-energy collisional dissociation; PTM, post-translational modification.

**Table 2:** Major PTM databases and their key features (all links accessed on 1 November 2025).

Database	PTM Focus	Key Features	URL
PTM-specific databases			
PhosphoSitePlus [51]	Phos, Ub, Acet, Methyl	Provides regulatory sites and PTMVars data linked to diseases and cancers.	https://www.phosphosite.org/homeAction.action
O-GlycBase [52]	O-gly, C-gly	Prediction tools for O-glycosylation sites based on neural network models.	https://services.healthtech.dtu.dk/datasets/OglycBase
UniCarbKB [53]	Gly	Integration with structural and experimental glycan databases; GlycoMod tool integration for predicting oligosaccharide structures.	http://unicarbkb.org
UbiProt [54]	Ub	Structured protein entry in block format for easy retrieval; detailed ubiquitylation features.	http://ubiprot.org.ru *
PHOSIDA [55]	Phos	Phosphosite predictor trained on >5000 high-confidence sites; motif searching and matching for user-generated or kinase motifs.	http://www.phosida.com *
Phospho.ELM [56]	Phos	Available structural disorder/order and accessibility information; conservation score visualisation with multiple sequence alignment.	http://phospho.elm.eu.org *
PhosphoPep [57]	Phos	Conservation analysis across species; mass spectrometric assays for quantification.	http://www.phosphopep.org
PhosPhAt [58]	Phos	Two search strategies: querying experimental data or phosphorylation site prediction.	http://phosphat.mpimpgolm.mpg.de *
Curated comprehensive databases			
UniprotKB [59]	Multiple PTMs	Machine learning-assisted curation for paper selection and data extraction; automatic annotation generation.	http://www.uniprot.org
neXtProt [60]	Multiple PTMs	Peptide uniqueness checker for identifying unique, pseudo-unique, or non-unique peptides, considering splicing and variants; in silico protein digestion tool for identifying proteases used in MS analysis.	https://www.nextprot.org
Integrative databases			
PTMcode2 [61]	Multiple PTMs	Residue co-evolution and proximity-based methods for predicting functional PTM associations; PTM propagation to orthologous proteins for understudied organisms.	https://ptmcode.embl.de
dbPTM [62]	Multiple PTMs	Advanced search and visualisation tools for efficient querying and data analysis; functional annotations and disease associations, highlighting cancer-specific PTM regulations.	https://biomics.lab.nycu.edu.tw/dbPTM
iPTMnet [63]	Phos, Ub, Acet, Methyl, Gly, SNO, SUMO, Myr	Integrative bioinformatics approach combining text mining, data mining, and ontological representation; captures enzyme-substrate relationships and PTM conservation; tools for search, retrieval, and visual analysis.	http://proteininformationresource.org/iPTMnet

Abbreviations: Acet, acetylation; C-gly, C-glycosylation; Gly, glycosylation; Methyl, methylation; Myr, myristoylation; O-gly, O-glycosylation; Phos, phosphorylation; SNO, S-nitrosylation; SUMO, SUMOylation; Ub, ubiquitination. * Currently inaccessible.

**Table 3:** Comparison of computational tools for PTM analysis (all links accessed on 1 November 2025).

Tool	Availability	Compatible Search Engines	PTM Focus	Implementation Method	Key Points of Method	URL
PTM localisation refinement tools						
Mascot Delta Score [74]	Commercial	Mascot	Phos	Difference score	Calculated based on the difference between the highest and second-highest Mascot ion scores for alternative phosphorylation site localisations of the same peptide sequence.	https://www.matrixscience.com
SLIP Score [75]	Open source	ProteinProspector	Phos	Difference score	Calculated by comparing the probability or expectation values between the best and next best site assignments for the same peptide, with the difference converted into a Log10-based integer score.	https://prospector.ucsf.edu/prospector/mshome.htm
ASCORE [71]	Open source	SEQUEST, Mascot	Phos	Peak probability score	Calculated by subtracting the cumulative binomial probabilities of the top two site candidates, measuring the likelihood of matching site-determining ions by chance.	http://Ascore.med.harvard.edu *
PTM Score [67]	Open source	Andromeda	Any PTMs available by the database used.	Peak probability score	Calculated using a binomial distribution formula to score MS/MS spectra, dividing the spectrum into 100 Th mass ranges and prioritising peaks by intensity.	https://www.maxquant.org
PhosCalc [76]	Open source	Any (uses DTA input files)	Phos	Peak probability score	Calculated based on successful matches of theoretical b and y ions, with the probability score.	http://www.ayeaye.tsl.ac.uk/PhosCalc *
PhosphoRS [77]	Open source	Search engines within the Proteome Discover suite	Phos	Peak probability score	Calculated using random matches between theoretical and experimental fragment ions using a cumulative binomial distribution.	https://ms.imp.ac.at/?goto=phosphors
P-brackets [78]	Open source	SEQUEST, Mascot	Phos	Ion pair-based score	Calculated using phosphorylation brackets, with the P-bracket score determined by the number of complementary product ion pairs that localise a phosphorylation event to a unique site.	http://proteingoggle.tongji.edu.cn *
LuciPHOr2 [79]	Open source	Any (uses pepXML input files)	Any PTMs of a fixed mass	Peak probability score	Calculated based on a probability model of peak intensity and mass accuracy, with dynamic training for each dataset and user-defined parameters for PTM analysis.	https://luciphor2.sourceforge.net
SLoMo [80,81]	Open source	Any (uses pepXML input files)	Phos, Acet, Ox, Carba, Deam	Peak probability score	Calculated based on the ASCORE algorithm with enhancements: user-defined modifications, customisable ion sets, and inclusion of hydrogen transfer ions.	http://massspec.bham.ac.uk/slomo *
PTMiner [82]	Open source	Any (requires tab-delimited files or outputs from pFind, SEQUEST, or MSFragger)	Phos, Acet, Ox, Meth, Deam	Posterior probability score	Calculated by combining prior probabilities from the MSFS vector with conditional probabilities from an intensity distribution model fitted on matched peaks of unmodified PSMs.	http://fugroup.amss.ac.cn/software/ptminer/ptminer.html
PTMProphet [70]	Open source	SEQUEST, Mascot, X!Tandem, Comet, ProteinProspector, MS-GF+, MSFragger	Any PTMs	Peak probability score	Calculated based on observed intensities and peaks, applying a Bayesian framework with renormalised probabilities to reflect the likelihood of modification at each site.	http://www.tppms.org/tools/ptm
MetaMorpheus [73]	Open source	Any (requires Thermo.raw, .mzML in centroid mode, or .mgf input file formats)	Any PTMs available by the database used.	Multi-notch search	First multi-notch search: Limiting mass differences to preselected values, improving specificity and reducing search time. Final limited multi-notch search: Accounting for precursor mass deisotoping errors and identifying co-isolated peptides, enhancing peptide and PTM identification.	https://smith-chem-wisc.github.io/MetaMorpheus
Bottom-up proteomics search engine with PTM support						
Byonic [72]	Commercial	No additional search engine needed	Any PTMs, whether present or absent in the database used.	IMP-ptmRS node	Three major features: Modification fine control allows for simultaneous search for multiple PTMs without a combinatorial explosion. Wildcard search enables a search for unanticipated modifications. Glycopeptide search identifies glycosylated peptides without predefined sites or masses.	http://www.proteinmetrics.com
Top-down proteomics search engines						
ProSight PD	Commercial	No additional search engine needed	Any proteoforms	ProSightPD nodes	Four core ProSightPD nodes: Feature Detector nodes perform spectral deconvolution using sliding window with Xtract or KDecon, measuring deconvoluted features and quantitation traces. Search nodes search assigned databases for protein identification and characterisation. cRAWler nodes deconvolute fragmentation spectra. ProSightPD Consensus nodes handle tasks ranging from grouping redundant PrSMs into proteoforms to assigning PFR accessions.	https://www.proteinaceous.net/prosightpd
TopPIC [83]	Open source	No additional search engine needed	Any proteoforms	PrSM processing algorithm and MIScore	Three-step algorithm for proteoform identification (core): (1) Protein filtering, (2) Spectral alignment, and (3) PrSM E-value computation. MIScore (optional): A Bayesian model-based method for characterising modifications explaining unknown mass shifts in PrSMs.	https://www.toppic.org/software/toppic/index.html
pTop [84]	Open source	No additional search engine needed	Any proteoforms	Sequence-tag-based search and dynamic programming algorithm	pParseTD: Potential precursor detection using SVM, followed by deconvolution and deisotoping of MS/MS spectra. Proteoform candidate retrieval: (1) Extract sequence tags and search against the protein database index, (2) Generate candidate modifications from the mass difference between the precursor and the protein. Modification localisation and proteoform ranking using the pDAG algorithm to identify the k-best paths.	http://pfind.ict.ac.cn/software/pTop/index.html *
MSPathFinder [85]	Open source	No additional search engine needed	Any proteoforms	Sequence-graph approach	ProMex: LC-MS feature-finding algorithm. MSPathFinder: (1) Sequence graph construction, (2) Proteoform scoring against MS/MS spectra through graph searching, and (3) FDR estimation.	https://github.com/PNNL-Comp-Mass-Spec/Informed-Proteomics

Abbreviations: Acet, acetylation; Carba, carbamidomethyl; Deam, deamidation; Meth, methylation; Ox, oxidation; Phos, phosphorylation. * Currently inaccessible.

**Table 4:** Exemplary studies of non-standard PTMs in cardiovascular research using MS-based proteomics techniques. All studies published since 2021 in a journal of impact factor bigger than 3 were included if they deployed discovery MS-based proteomics in a sample size bigger than 10.

Study	PTM	Key Proteins Involved	MS-Proteomics Technique	Main Finding
Yang et al., 2025 [99]	Ubiquitinoylation	SERCA2, SIRT2	Co-immunoprecipitation combined with MS	Succinylation of SERCA2a, controlled by SIRT2, promotes its ubiquitinoylation and degradation by proteasomes in sepsis-induced heart dysfunction.
Li et al., 2025 [100]	S-nitrosylation	HBb, Trx, GSNOR	TMT-labelled LC-MS/MS	Identification of S-nitrosylated proteins associated with HFpEF.
Wu et al., 2022 [101]	Malonylation	IDH2	Label-free LC-MS/MS	Malonylated IDH2 is downregulated cardiac hypertrophy.
Theofilatos et al., 2023 [2]	γ-Carboxylation	MGP, F2, GAS6, PROC, PROZ	TMT-labelled LC-MS/MS, Targeted Proteomics (Multiple Reaction Monitoring, MRM)	γ-carboxylated proteins are upregulated in female, asymptomatic and calcified plaques and drive carotid plaque clustering into subgroups with different outcome trajectories.
Yang et al., 2023 [102]	Lysine β-hydroxybutyrylation	MMP2, ALAD, EPB42	Label-free LC-MS/MS	The Kbhb-modified proteins upregulated in aged hearts were primarily detected in energy metabolism pathways and localised in the mitochondria.
Liu et al., 2025 [103]	Lysine β-hydroxybutyrylation	COL1A1	Label-free LC-MS/MS	Lysine β-hydroxybutyrilated COL1A1 was downregulated in metabolic syndrome induced restenosis.
Wang et al., 2025 [104]	Lactylation	SERPINA3K, SERPINA3	Label-free LC-MS/MS	The protective role of Serpina3k and Serpina3 lactylation though their secretion from ischemia-reperfusion-stimulated fibroblasts to protect cardiomyocytes from reperfusion-induced apoptosis.
Hasman et al., 2023 [105]	Oxidation	FLNA	Label-free LC-MS/MS	Oxidated FLNA is interacting cell–cell communication, neutrophil degranulation, and smooth muscle cell contraction.
Bagwan et al., 2021 [98]	150 PTMs	MYH6, MYH7, PLN, TNNI3, MYBPC3, SCN5A, RYR2, CACNA1C	Label-free LC-MS/MS	Provided a resource of more than 150 PTMs in human hearts.

## Data Availability

No new data were created or analysed in this study. Data sharing is not applicable to this article.

## Abbreviations

The following abbreviations are used in this manuscript:

CID	Collision-induced dissociation
CVD	Cardiovascular disease
ETD	Electron transfer dissociation
FLR	False localisation rate
GRP	Gla-rich protein
LC-MS/MS	Liquid chromatography–tandem mass spectrometry
lncRNA	Long non-coding RNA
MGP	Matrix Gla protein
MS	Mass spectrometry
*m*/*z*	Mass-to-charge ratio
ncRNA	Non-coding RNA
PTM	Post-translational modification
TMT	Tandem mass tag
